# Multi-flap Cheek Reconstruction: Merging Triple Advancement, Island Pedicle, and Rotation Flaps for Optimal Aesthetic and Functional Outcomes After Mohs Micrographic Surgery

**DOI:** 10.7759/cureus.72139

**Published:** 2024-10-22

**Authors:** Jena C Jacobs, Mario J Sequeira

**Affiliations:** 1 Dermatology, A.T. Still University, Kirksville, USA; 2 Dermatology, Brevard Skin and Cancer Center, Rockledge, USA; 3 Dermatology, University of Miami Miller School of Medicine, Miami, USA

**Keywords:** advancement flap, burow's graft, cutaneous oncology, facial reconstruction, mercedes flap, mohs micrographic surgery (mms), mohs surgery, skin rotation flap, tripolar advancement flap, wound reconstruction

## Abstract

An 85-year-old white male presented with a basal cell carcinoma on the right cheek, treated with Mohs micrographic surgery, resulting in a 5.8 x 3.2 cm defect across multiple facial subunits. Given the defect's size and patient's age, options such as second-intention healing, primary closure, and skin grafting were deemed suboptimal. The cheek's anatomical complexity requires innovative surgical strategies for large defects. A multi-faceted surgical approach using adjacent tissues was employed: a triple advancement flap, an island pedicle, and a rotation flap to optimize aesthetic and functional outcomes. The defect was primarily closed using a triple advancement technique to minimize the tension and distortion at the lateral canthus and oral commissure. An island pedicle flap was then utilized to close the anterior part of the defect. Finally, a rotation flap and excision of a small standing skin cone allowed repair of the superior defect with minimal distortion of the lateral canthal skin. Key suturing strategies were implemented to maximize skin laxity and minimize aesthetic disruption. By integrating Mohs surgery, multiple flap techniques, and careful tension management, effective closure was achieved with excellent oncologic and aesthetic outcomes. This case underscores the importance of tailored surgical approaches in facial reconstructive surgery, particularly in elderly patients with significant aesthetic and functional considerations.

## Introduction

The cheek, the largest aesthetic unit of the face, is bordered medially by the nasolabial, melolabial, and mentofacial folds; laterally by the pinna and the angle of the mandible; superiorly by the infraorbital rim and zygomatic arch; and inferiorly by the lower border of the mandible [1]. The decision to perform a skin flap on the cheek must be weighed against the other possible repair options: 1) second-intention healing, 2) intermediate or complex primary side-to-side linear closures, or 3) skin grafts. This particular post-surgical wound was too large and prone to bleeding for the options of second intention healing or primary closure, respectively. While a split-thickness skin graft could cover the defect, it would likely result in color mismatch and suboptimal contour. A full-thickness skin graft (FTSG) of this size was at higher risk of necrosis due to its greater metabolic demands. Although a cervicofacial flap was considered, the extensive subcutaneous dissection required for its advancement and rotation was not ideal [2,3]. Similarly, a large neck transposition flap posed significant challenges for this elderly patient who was also on a daily aspirin regimen [4]. For large lesions (>3 cm), such as this one, a cervicofacial rotation flap should be considered [3,5]. For anteriorly based cervicofacial flaps, the lateral incision can extend to the hairline, along the preauricular area, and then down the neck (or chest even) where a back-cut is needed to facilitate tissue advancement [5]. Due to the size of our lesion, we designed our repair utilizing multiple techniques, all using adjacent tissue from the pre-auricular, neck, and cheek. We maximized the use of pliable tissue and minimized utilization of taut areas where the tension vectors would have distorted the normal anatomy.

## Case presentation

**Figure 1:**
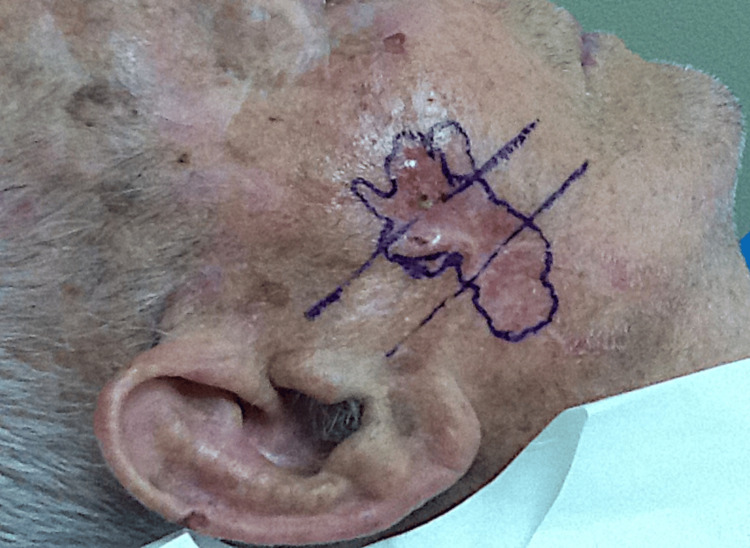
Pre-operative nodular Basal Cell Carcinoma measuring 5.8 X 3.2 cm

**Figure 2:**
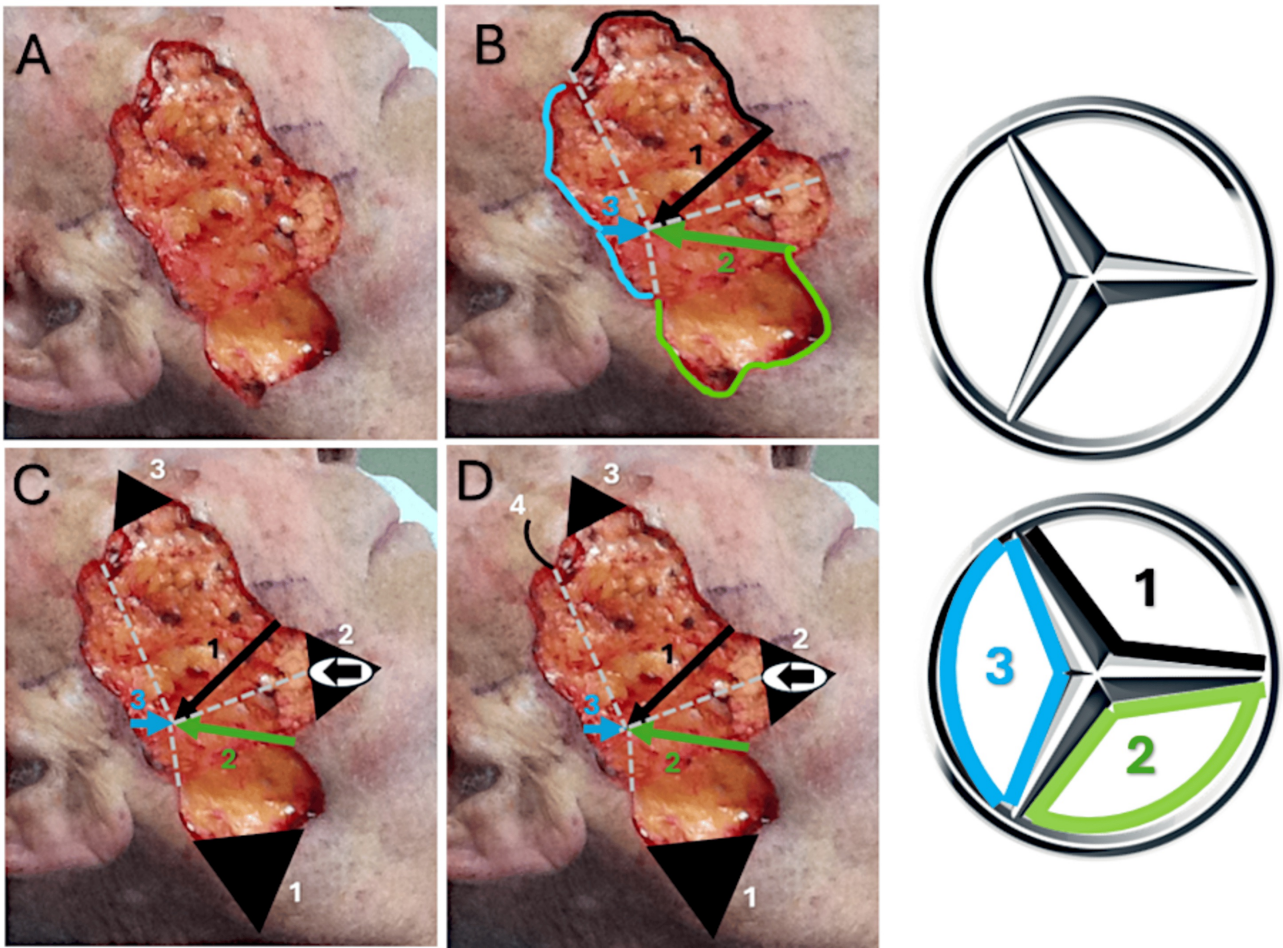
A: Mohs surgical defect (5.8 x 3.2 cm) after three stages to achieve clear margins. B: A multi-modal repair utilizing a triple advancement flap, island pedicle flap and rotation flap was designed. Skin advanced medially along three edges, steps indicated numerically. The first key subcutaneous 4-0 polyglactin 910 suture approximated the anterior and posterior wound edges side to side (1-2-3). C: 1, Burow’s full-thickness triangle excised; 2, Triangular island pedicle flap advanced posteriorly; 3, Full-thickness triangle excised. D: Superior rotation flap closed. See Mercedes logo to the right for shape reference.

**Figure 3:**
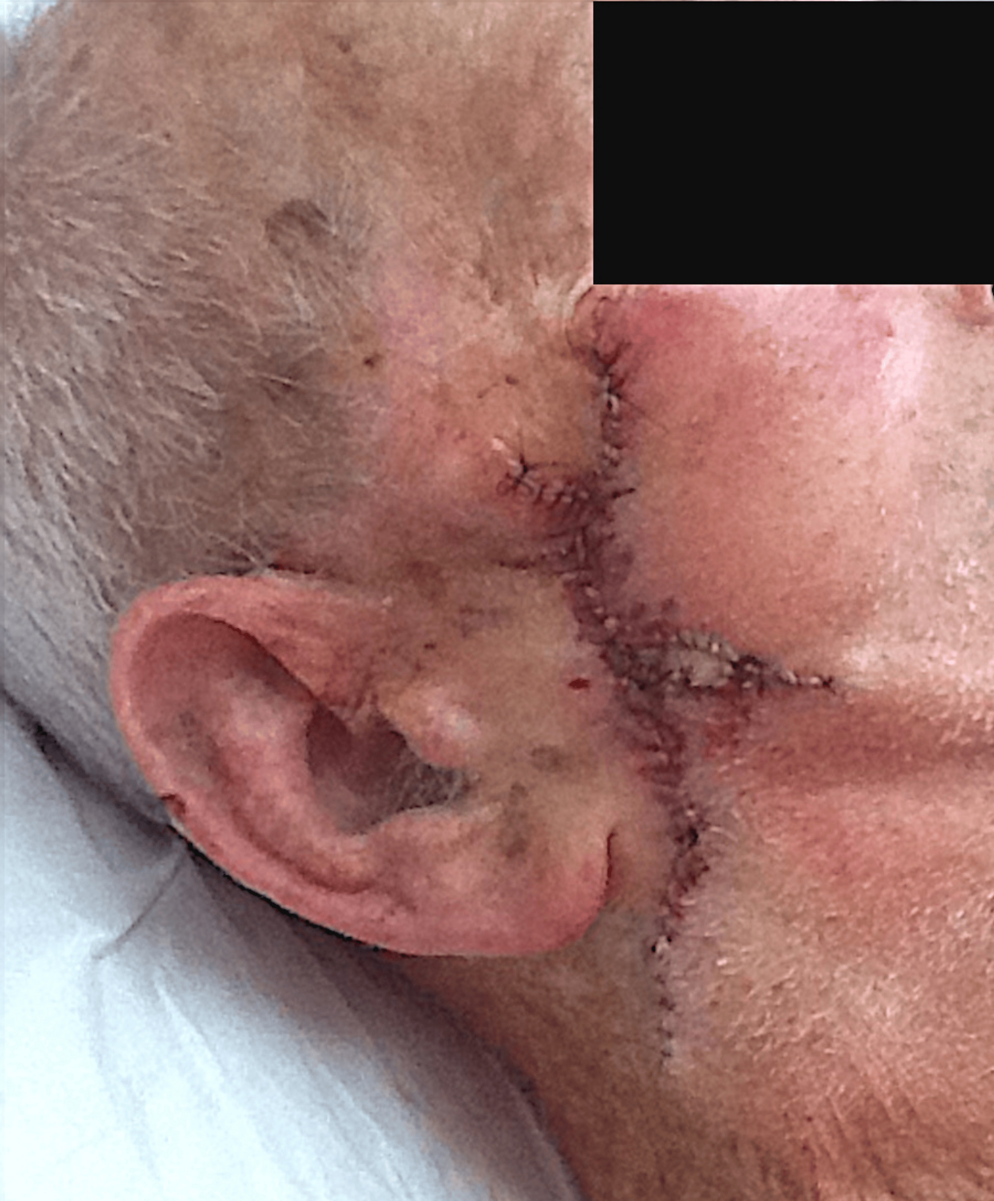
Immediate postoperative result. Flaps sutured into place.

An 85-year-old white male presented for treatment of an ulcerated nodular basal cell carcinoma of the right cheek (Figure 1). The tumor was removed with three stages of Mohs surgery resulting in a defect measuring 5.8 x 3.2 cm and involving the buccal, zygomatic, and parotido-masseteric subunits of the right cheek (Figure 2A). A multi-modal repair utilizing a triple advancement flap, island pedicle flap, and rotation flap was designed. After wide undermining, hemostasis was expeditiously achieved with electrocoagulation. Using fine-toothed forceps, the wound edges were gently pinched and approximated to gauge their best apposition paying close attention to the maximum recruitment of lax skin and the least distortion of the free edge of the oral commissure and lateral canthus. The skin was then advanced along three edges [4]. The first key subcutaneous 4-0 polyglactin 910 suture approximated the anterior and posterior wound edges side to side (1-2-3) (Figure 2B). The triple advancement created three areas of triangular standing skin cones (Figure 2C). First, a Burow’s full-thickness triangle was excised inferiorly from the standing skin cone labeled "1" (Figure 2C). This triangular tissue was not discarded but saved in the surgical tray on top of a saline-moistened gauze if needed for possible later use as a Burow’s graft. Secondly, the triangular standing skin cone on the anterior wound edge, labeled "2", created by the triple advancement of tissue was scored and undermined along its perimeter while remaining attached to its central subcutaneous pedicle. It was then advanced posteriorly (Figure 2C). The excess skin on the perimeter was gently trimmed and the required oval island pedicle flap sutured in place centrally where there was too much tension to approximate the wound edges primarily (Figure 3). If there had been limited mobility of the pedicle flap, we were prepared to use it as a contiguous Burow’s full-thickness graft for closure. The mid and inferior aspects of the defect were then closed with subcutaneous 5-0 polyglactin 910 sutures. Thirdly, our attention was then directed to the superior aspect of the wound to the final skin cone, labeled triangle "3", which was excised and discarded (Figure 2C). Finally, in this same region, a small rotation flap was designed to minimize distortion of the lateral canthal skin, and the tissue labeled "4" was rotated and secured with a subcutaneous 5-0 polyglactin 910 tip suture (Figure 2D, Figure 3). A total of four interrupted and three running epidermal 5-0 nylon sutures were then used to fully repair the rest of the wound (Figure 3). A clotting powder (hydrophilic polymer and potassium ferrate) was applied on areas of fine pinpoint bleeding along the mid aspects of the repair (grey appearance along mid-suture line). 

**Figure 4:**
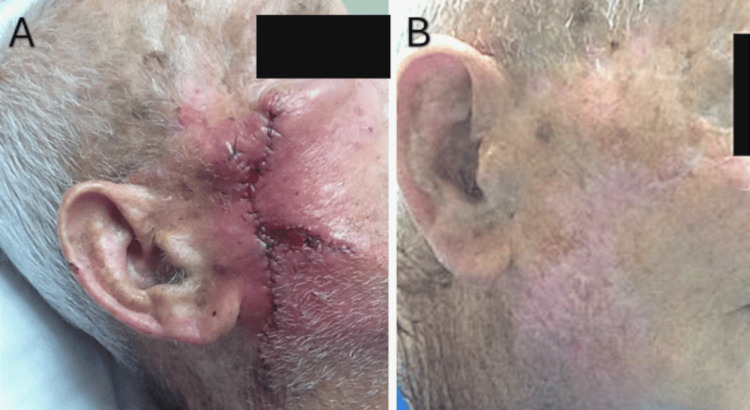
A: Nine days later at suture removal appointment, B: Eight-month follow-up

The patient returned nine days postoperatively for suture removal (Figure 4A) and again eight months later for follow-up and was cancer-free and satisfied with his surgical outcome (Figure 4B).

## Discussion

The surgical management of complex facial defects, particularly in the cheek, demands precise techniques that respect aesthetic units while ensuring functional integrity. In the present case, a combination of triple advancement flap, island pedicle flap, and rotational flap techniques were employed, each contributing uniquely to the reconstruction’s success.

The triple advancement flap, also known as the tripolar advancement flap, three-point advancement flap, or Mercedes flap, is an excellent reconstruction technique for facial defects that may result in distortion of facial anatomic structures via other common repair methods [6]. Although limited, the current literature suggests use of the triple advancement flap for large defects on the body, extremities, and scalp, with select reports on the temple and forehead [7]. Although rarely used, the triple advancement flap is versatile and particularly useful in facial reconstructive surgery due to its ability to mobilize large areas of skin with minimal tension and distortion of adjacent anatomical landmarks [6]. This method involves creating three separate flap advancements to cover the defect. The inherent benefit of this technique lies in its capacity to distribute the tension across multiple vectors, which minimizes the risk of cicatricial ectropion and other deformities associated with unilateral tension [6]. We feature a novel use of this technique for deficits located on the cheek, as this technique allowed for effective recruitment of adjacent lax skin, crucial in managing the large defect without altering the free edges of the oral commissure and lateral canthus.

The island pedicle flap, also known as a subcutaneous pedicle flap, was utilized to manage areas where primary closure was not feasible due to excessive tension. This flap type is characterized by its random pattern blood supply and can be mobilized over a considerable distance while maintaining robust vascularity [8]. It is typically utilized on the nasal tip, the nasal ala, the upper cheek, and the upper lip [8]. This flap was a crucial aspect of the success of this closure and was designed to take advantage of a standing skin cone created by the tissue movement of the prior triple advancement flap.

The rotational flap technique was specifically employed to minimize distortion of the lateral canthal skin, an area particularly prone to aesthetic and functional complications if altered [9]. Rotational flaps are curved incisions that allow skin and subcutaneous tissue to be mobilized based on a pivot point, redistributing skin from an area of redundancy to the defect [9]. This method was invaluable in the superior aspect of the wound, allowing for a seamless closure that maintained the natural curvature and position of the lateral canthus, thus preserving the patient’s facial symmetry, as was pertinent in this case.

The strategic combination of these flaps not only maximized the functional and cosmetic outcome but also illustrated the principle of using local tissues to maintain the harmony and natural contours of the face. The choice of technique in each specific area of the defect was dictated by the unique demands for mobility, blood supply, and minimal donor site morbidity, as well as the overall need to reduce the visible impacts of surgery. In this case we present the successful integration of these techniques, underscoring the importance of a tailored approach in facial reconstructive surgery, especially in complex cases involving elderly patients with additional systemic considerations.

Some additional reconstruction considerations from the authors are as follows: For large cheek repairs, it is crucial to position key sutures to maximize the use of available lax skin while minimizing distortion of the lateral canthus and oral commissure. The placement of subcutaneous sutures may need to be adjusted to redirect tension and prevent distortion or unfavorable tension vectors. Additionally, when considering the excision of a Burow’s triangle, starting the incision on the perimeter and evaluating the central subcutaneous pedicle's mobility is essential. This approach allows for the possibility of advancing the pedicle as an island flap or, if necessary, utilizing it as a full-thickness skin graft if mobility is limited.

There are potential limitations and complications of the multi-modal repair approach. Bleeding, infection, and scarring are common risks with any procedure that breaks the skin, and advancement flaps are no exception. These flaps are often used to close large wounds, but bleeding during surgery is the most common issue, especially in Mohs micrographic surgery [6]. Reducing the risk of bleeding starts before surgery with a careful review of medications, including over-the-counter ones. For patients on blood thinners, it’s generally recommended they stay on their medication since the risk of a blood clot is greater than the chance of bleeding during surgery [6]. If there’s a chance of significant bleeding after surgery, placing a drain at the base of the flap can help prevent a blood clot (hematoma), which could affect how well the flap heals [6]. Another possible limitation is the necrosis of the pedicle island. It is imperative to strategically suture so this island flap can still get proper blood flow and nutrients. 

## Conclusions

Repairing large wounds often requires a strategic combination of modalities to achieve optimal results and utilizing multiple flaps across different regions of the wound defect can enhance the repair process. The triple advancement flap is a relatively simple, versatile flap with scope well beyond that currently described in the literature. We recommend broadening the utility of this flap, considering it for nasal sidewall and nasal root defects among other reconstructive options. Additionally, we believe this repair method has utility in many other facial regions and hope these cases prompt broader adaptation and additional novel locations. In combination with the other two modalities, we show that the combination of these flaps may be a technically and aesthetically superior method of repair for large defects in the anterior cheek.
